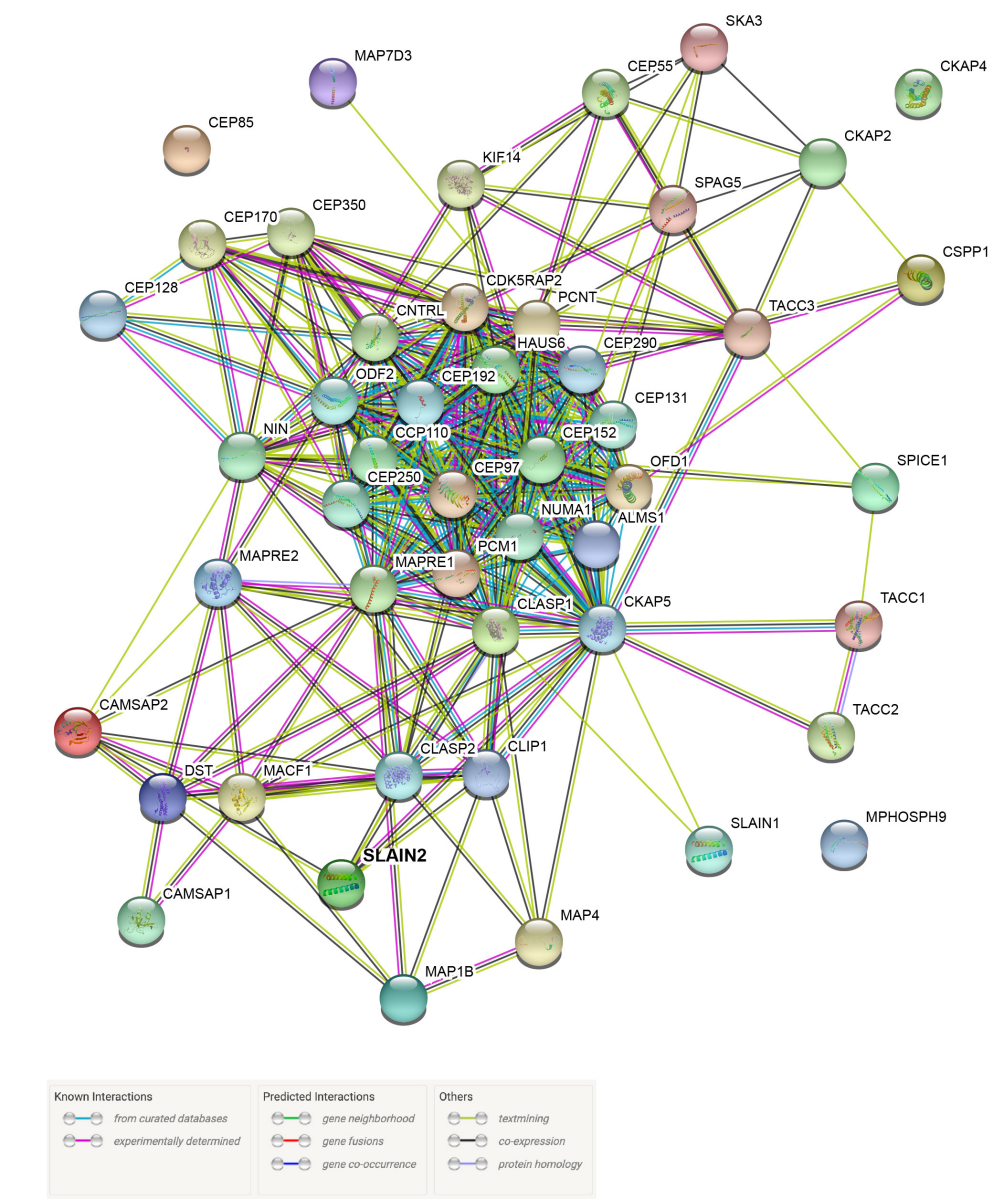# Proximity mapping of the microtubule plus-end tracking protein SLAIN2 using the BioID approach

**DOI:** 10.3906/biy-2002-12

**Published:** 2020-04-02

**Authors:** Elif Nur FIRAT-KARALAR

**Affiliations:** 1 Department of Molecular Biology and Genetics, Faculty of Science, Koç University, İstanbul Turkey

**Keywords:** SLAIN2, microtubules, proximity mapping

## Abstract

The centrosome is the main microtubule-organizing center of animal cells, which plays key roles in critical cellular processes ranging from cell division to cellular signaling. Accordingly, defects in the structure and function of centrosomes cause various human diseases such as cancer and primary microcephaly. To elucidate the molecular defects underlying these diseases, the biogenesis and functions of the centrosomes have to be fully understood. An essential step towards addressing these questions is the identification and functional dissection of the full repertoire of centrosome proteins. Here, we used high-resolution imaging and showed that the microtubule plus-end tracking protein SLAIN2 localizes to the pericentriolar material at the proximal end of centrioles. To gain insight into its cellular functions and mechanisms, we applied in vivo proximity-dependent biotin identification to SLAIN2 and generated its proximity interaction map. Gene ontology analysis of the SLAIN2 interactome revealed extensive interactions with centriole duplication, ciliogenesis, and microtubule-associated proteins, including previously characterized and uncharacterized interactions. Collectively, our results define SLAIN2 as a component of pericentriolar material and provide an important resource for future studies aimed at elucidating SLAIN2 functions at the centrosome.

## 1. Introduction

The centrosome is the main microtubule-organizing center of animal cells, which play skey roles in critical cellular processes ranging from cell division to cellular signaling (Chavali et al., 2014). The centrosome consists of two cylindrical microtubule-based structures termed centrioles and associated pericentriolar material, which supports microtubule nucleation, polymerization, and stability (Bettencourt-Dias and Glover, 2007; Luders and Stearns, 2007; Nigg and Raff, 2009). In cycling cells, centrosomes organize the interphase microtubule network, which is required for vesicular trafficking, cell migration and cell shape (Luders and Stearns, 2007). In dividing cells, centrosomes form the mitotic spindle, which mediates equal segregation of genetic material to daughter cells (Nigg and Raff, 2009). In some cycling cells and most quiescent noncycling cells, one of the centrioles forms the basal body that nucleates the microtubule axoneme of the primary cilium. The primary cilium is a nonmotile sensory organelle, which serves as the nexus for growth factor and mechano-sensing signaling pathways important in development and tissue homeostasis (Malicki and Johnson, 2017; Mirvis et al., 2018). In addition, specialized multiciliated epithelial cells such as trachea and fallopian tube have many motile cilia on their surface that mediates liquid movement, and sperm cells have a single motile cilium that is required for their motility. 

Centrosome and cilium dysfunction are associated with a variety of human diseases including cancer and ciliopathies (Hildebrandt et al., 2011; Nigg et al., 2014). The association between centrosomal abnormalities and cancer was first observed in the late 1800s; indeed it was one of the first cell biological defects noted in cancer cells (Boveri 2008). The most obvious defect is that cancer cells often have supernumerary centrosomes, which is associated with genome instability due to chromosome attachment errors (Bettencourt-Dias et al., 2011). Moreover, recent studies demonstrated that extra centrosomes disrupts ciliary signaling and epithelial organization through formation of extra cilia and promote invasive behavior through activation of Rac-mediated actin polymerization (Nigg and Holland, 2018). Given their sensory roles in developmental signaling pathways, defects in ciliogenesis and cilium function results in genetic diseases called ciliopathies, which are characterized by a broad spectrum of anomalies in multiple organ systems (Hildebrandt et al., 2011; Braun and Hildebrandt, 2017). To better understand these disease connections and develop new diagnostic and therapeutic approaches, the mechanisms underlying the biogenesis and functions of centrosomes and cilia have to be fully understood. An essential step towards addressing this question is the identification of the full repertoire of proteins that localize to centrosomes and cilia and the subsequent functional and biochemical dissection of these proteins. 

Over the years, a combination of proteomic, transcriptomic, bioinformatics studies, and genetic screens has identified many proteins that localize to the centrosome. The proteomic analysis of the human interphase centrosome by combining centrosome purifications with protein correlation profiling by Andersen 2003 (Andersen et al., 2003) identified 108 proteins; the genome-wide RNA interference by Dobellaere (2008) identified 32 proteins, the proteomic profiling of the mitotic Drosophila centrosome by Muller (2005)  identified 251 proteins and the proteomic analysis of the mammalian sperm centrioles by Firat-Karalar (2014) identified 364 proteins (Dobbelaere et al., 2003; Muller et al., 2005; Firat-Karalar et al., 2014). A key advance in expanding these proteomic lists and probing the interactions among these proteins is the application of the proximity-labeling approaches to the centrosome (Firat-Karalar et al., 2014; Firat-Karalar and Stearns 2015). BioID proximity-labeling makes use of promiscuous biotin ligases to identify the proximity partners within 10 nm diameter of the proteins of interest (Roux et al., 2013). The proximity mapping of 58 centrosome proteins generated a topology protein interaction network comprising more than 7000 interactions (Gupta et al., 2015). The advantage of the proximity labeling approaches is their ability to identify transient and insoluble interactions of bait proteins, which are not accessed before using traditional pulldowns (Lambert et al., 2014). Although these studies together revealed a nearly complete centrosome proteome, the functions and mechanisms of many of centrosome proteins still remain poorly understood. Among these proteins, we chose SLAIN2 for further characterization due to its reported functions in microtubule dynamics and mesenchymal cell invasion as well as its link to colorectal cancer (van der Vaart et al., 2011; van der Vaart et al., 2012; Bouchet et al., 2016; Zhuang et al., 2019). 

SLAIN2 was first identified in the purified centrosome fractions of mammalian lymphoblasts in a high-throughput mass spectrometry study and subsequently GFP-SLAIN2 was shown to localize to centrosomes (Jakobsen et al., 2013). SLAIN2 was later identified as a new microtubule plus-end tracking protein (+TIP) that forms a complex with end-binding proteins (EBs), cytoplasmic linker proteins (CLIPs) and ch-TOG, and promotes processive microtubule growth in interphase cells (van der Vaart et al., 2011). Moreover, SLAIN2 and CLASP1 together suppress catastrophes and are essential for mesenchymal cell invasion in 3D culture and in a mouse cancer model (Bouchet et al., 2016). Supporting its link to cancer, the long noncoding RNA MALAT1 was shown to regulate miR-106b-5p expression to promote the invasion and metastasis of colorectal cancer by enhancing the mobility of microtubules (Zhuang et al., 2019). Although these studies provided new insight into the control of microtubule plus end dynamics in cells and in cancer, the centrosomal functions and mechanisms of SLAIN2 remain unknown. Here, we used high-resolution microscopy, BioID proximity-mapping, and gene ontology analysis to determine the cellular localization of SLAIN2 and its interactions. The results of our study identified SLAIN2 as a proximal component of centrosomes and revealed extensive interactions with a wide range of centrosome proteins including the ones implicated in centriole duplication and cilium assembly. Collectively, these results provide new insight into the centrosomal functions of SLAIN2 and the molecular defects underlying cancer associated with deregulation of SLAIN2. 

## 2. Materials and methods

### 2.1. Plasmids 

Full-length cDNA of SLAIN2 (GenBank Accession. NM_020486.1) was obtained from DF/HCC DNA Resource Core (Harvard Medical School, MA). pCMVt7-mh-Cep152 (GenBank Accession no. NM_001194998) was previously described and used for expression of myc-Cep152 (Hatch et al., 2010). The ORF of SLAIN2 was amplified by PCR and cloned into pDONR221 using Gateway cloning (Invitrogen). Subsequent Gateway recombination reactions using pLVPT2-V5-BirA* provided by Christian Hoerner (Stanford University, Stanford, CA) was used to generate V5-BirA*-SLAIN2. All constructs were verified by Sanger sequencing.

### 2.2. Cell culture and transfection

Human bone osteosarcoma (U2OS, ATCC, HTB-96) and human embryonic kidney (HEK293T, ATCC, CRL-3216) cells were cultured in DMEM supplemented with 10% FBS and 1% penicillin/streptomycin at 37 °C and 5% CO2. U2OS and HEK293T cells were tested for mycoplasma contamination by MycoAlert Mycoplasma Detection Kit (Lonza). For microtubule depolymerizaton experiments, cells were seeded on glass coverslips in 24-well plates and treated with 10 μg/mL nocodazole (Sigma-Aldrich) diluted from 10 mM stock solution in DMSO for 1 h at 37 °C. DMSO was diluted at 1:1000 and used as vehicle control. U2OS cells were transfected with the plasmids using Lipofectamine 2000 according to the manufacturer’s instructions (Invitrogen). HEK293T cells were transfected with the plasmids using 1 μg/μl polyethylenimine, MW 25 kDa (PEI, Plysciences, Warrington, PA).

### 2.3. Lentivirus production and cell transduction

HIV-derived recombinant lentivirus expressing V5-BirA* or V5-BirA*-SLAIN2 were generated using the pLVPT-V5-BirA* and pLVPT-V5-BirA*-SLAIN2 lentiviral transfer vectors, respectively. HEK293T cells were grown to 70–80% confluency and cotransfected with the pCMVDR8.74 packaging vector, pMD2.VSVG envelope vector (Dull et al., 1998) and transfer vectors using 1 µg/µL PEI. Medium was changed 6 h posttransfection and lentiviral supernatant lentiviral supernatant was harvested after 48 h and passed through a 0.45-µm filter. The viral supernatant was titered by transducing cells with varying volumes of virus. For transduction, 2 × 104 U2OS cells were seeded on 12-well tissue culture plates and transduced with lentivirus with an approximate multiplicity of infection (MOI) of 1 in 2 consecutive days. Transduction efficiency was determined by immunofluorescence experiments. U2OS and HEK293T clones stably expressing V5-BirA*-SLAIN2 were expanded and used in immunofluorescence and proximity labeling experiments. 

### 2.4. Streptavidin pulldown experiments

HEK293T stably expressing V5-BirA*-SLAIN2 or V5-BirA* were grown in 5 × 15 cm dishes. At 70–80% confluency, cells were incubated with complete medium supplemented with 50 μM biotin. After 18 h of incubation with biotin, the cells were scraped and lysed at 25 °C in lysis buffer (50 mM Tris, pH 7.4, 500 mM NaCI, 0.4% SDS, 5 mM EDTA, 1 mM DTT, 2% Triton X-100, protease inhibitors) and sonicated. After adding an equal volume of 4 °C 50 mM Tris (pH 7.4) to the extract, insoluble material was pelleted by centrifugation at 14,000 rpm for 20 min. The soluble supernatants were incubated with Streptavidin agarose beads (Invitrogen) overnight at 4 °C, followed by washing twice in wash buffer 1 (2% SDS in dH2O), once with wash buffer 2 (0.2% deoxycholate, 1% Triton X-100, 500 mM NaCI, 1 mM EDTA, and 50 mM Hepes, pH 7.5), once with wash buffer 3 (250 mM LiCI, 0.5% NP-40, 0.5% deoxycholate, 1% Triton X-100, 500 mM NaCI, 1 mM EDTA and 10 mM Tris, pH 8.1) and twice with wash buffer 4 (50 mM Tris, pH 7.4, and 50 mM NaCI). After validation of successful pulldown by Western blotting using 10% of the sample, 90% of the remaining sample was washed twice in 50 mM NH4HCO3 and analyzed by mass spectrometry. 

### 2.5. Mass spectrometry experiments and data analysis 

Biotinylated proteins were digested following an on-bead tryptic digestion protocol and peptides were analyzed by online C18 nanoflow reversed-phase nano liquid chromatography (Dionex Ultimate 3000 RS LC, Thermo Scientific) combined with orbitrap mass spectrometer (Q Exactive Orbitrap, Thermo Scientific). Samples were separated in an in-house packed 75 μm i.d. × 23 cm C18 column (Reprosil-Gold C18, 3 μm, 200 Å, Dr. Maisch) using 75-min linear gradients from 5–25%, 25–40%, 40–95% acetonitrile in 0.1% formic acid with 300 nL/min flow for 90 min. The scan sequence began with an MS1 spectrum (Orbitrap analysis; resolution 70,000; mass range 400–1500 m/z; automatic gain control (AGC) target 1e6; maximum injection time 32 ms). Up to 15 of the most intense ions per cycle were fragmented and analyzed in the orbitrap with Data Dependent Acquisition (DDA). MS2 analysis consisted of collision-induced dissociation (higher-energy collisional dissociation (HCD)) (resolution 17,500; AGC 1e6; normalized collision energy (NCE) 26; maximum injection time 85 ms). The isolation window for MS/MS was 2.0 m/z.

Raw files were processed with Thermo Proteome Discoverer 1.4 .Carbamidomethylation of cysteine was used as fixed modification and acetylation (protein N-termini) and oxidation of methionine were used as variable modifications. Maximal two missed cleavages were allowed for the tryptic peptides. The precursor mass tolerance was set to 10 ppm and both peptide and protein false discovery rates (FDRs) were set to 0.01. The database search was performed against the human Uniprot database (release 2016). The database also had sequence for E. coli BirA-R118G appended to it.

Data for SLAIN2 BioID experiments were derived from three biological replicates. Control data were derived from 7 independent experiments of HEK293T cells stably expressing V5-BirA*. To compare data across different runs, the normalization of spectral counts (Zybailov et al., 2006) was applied with the exception of dividing normalized spectral counts to protein length. Briefly, spectral count for each protein was normalized against the sum of spectral counts in a particular run. Normalized spectral factors for each protein were multiplied by 100 for presentation purposes. To define the nonspecific interactions, the ration of spectral abundance factor of each protein was divided to the one in control if protein was identified in control datasets. A protein was considered a contaminant if the ratio was greater than 2. Additionally, proteins that were estimated as mass spectrometry contaminants by the contaminant repository for affinity purification-mass spectrometry data (Mellacheruvu et al., 2013) were filtered out as contaminant proteins if they were identified in more than 85 out of 411 experiments. Finally, we only accounted for proteins that were identified in at least 2 out of three independent experiments and that have a spectral count greater than 4. The numbers presented in Figure 3 is average spectral abundance factors of three independent experiments. Interaction networks were visualized in Cytoscape 3.7.1 (Shannon et al., 2003), and graphically edited in Adobe Illustrator 24.0.1. Gene ontology (GO) analysis were performed with the indicated proteins using the EnrichR web server (Kuleshov et al., 2016)

### 2.6. Antibodies

Anti-PCM1 antibody was generated and used for immunofluorescence as previously described (Firat-Karalar et al., 2014). Antibodies used for immunofluorescence experiments were rabbit anti-SLAIN2 (Proteintech, Cat#13908-1-AP) at 1:1000, mouse anti-gamma-tubulin (GTU-88; T5326; Sigma-Aldrich) at 1:4000, mouse anti alpha tubulin (Sigma, DM1A) at 1:1000, mouse anti-centrin 3 (Abnova; clone 3E6) at 1:750) and mouse anti-V5 (Thermo Scientific; R960-25) at 1:500. AlexaFluor 488-, 568-, or 633-coupled secondary antibodies (Life Technologies) were used at 1:2000. Nuclei was stained with 4-,6-diamidino-2-phenylindole (DAPI) at 1 µg/mL. Antibodies used for western blotting were rabbit anti-SLAIN2 (Proteintech, Cat#13908-1-AP) at 1:1000 and anti-p38 (Santa Cruz Biotechnology) at 1:1000. IRDye680- and IRDye 800-coupled secondary antibodies were used at 1:10,000 for western blotting experiments (LI-COR Biosciences).

### 2.7. Immunofluorescence and microscopy

Cells were grown on coverslips were washed two times with PBS and fixed in ice cold methanol at –20 °C for 10 min. After rehydration in PBS, cells were blocked with 3% BSA (Capricorn Scientific, Cat. # BSA-1T) in PBS + 0.1% Triton X-100 followed by incubation with primary antibodies in blocking solution for 1 h at room temperature. Following three washes with PBS, the cells were incubated with Alexa-coupled secondary antibodies and DAPI. The cells were washed three times with PBS and mounted using Mowiol mounting medium containing N-propyl gallate (Sigma-Aldrich). Fluorescence images were acquired using a HyVolution confocal scanning microscope equipped with HC PL APO CS2 63× 1.4 NA oil objective with 0.3 µm step size and 4 µm stack size in 1024 × 1024 pixel format. The z-stacks were used to assemble maximum-intensity projections. For higher resolution imaging of SLAIN2 at the centrosome, fluorescence signals were detected by HyD detectors and processed with Huygens Essential software (Scientific Volume Imaging). Image processing was performed using ImageJ (National Institutes of Health, Bethesda, MD). 

### 2.8. Cell lysis and western blotting

Cell extracts were prepared by lysing cells in 50 mM Tris (pH 7.6), 150 mM NaCI, 1% Triton X-100 and protease inhibitors for 30 min at 4 °C followed by centrifugation at 14,000 rpm for 15 min. The protein concentration of lysates was determined with the Bradford solution (Bio-Rad Laboratories, CA, USA) using bovine serum albumin as a standard. For western blotting, cell extracts were resolved on 10% acrylamide sodium dodecyl sulphate polyacrylamide gel electrophoresis (SDS-PAGE) using the Mini-Protean system (Bio-Rad Laboratories, CA, USA) followed by transfer onto nitrocellulose membranes using the Mini Transblot wet transfer system (Bio-Rad Laboratories, CA, USA). Membranes were incubated in blocking solution (PBS+5% milk, 0.1%Tween-20) for 1 h at room temperature, incubated with primary antibodies overnight at 4 °C and washed three times with PBS+0.1%Tween-20 (PBST). After secondary antibody incubation for 1 h at room temperature and washing three times with PBST, the blots were scanned using the LI-COR Odyssey® scanner and software (LI-COR Biosciences) at 169 µm. 

### 2.9. Statistical analysis

Statistical results, average, and standard deviation values were computed and plotted by using Prism (GraphPad, La Jolla, CA). Two-tailed t-tests and ANOVA were applied to compare the statistical significance of the measurements. 

## 3. Results

### 3.1. SLAIN2 localizes to the centrosome throughout the cell cycle

**Figure 1:**
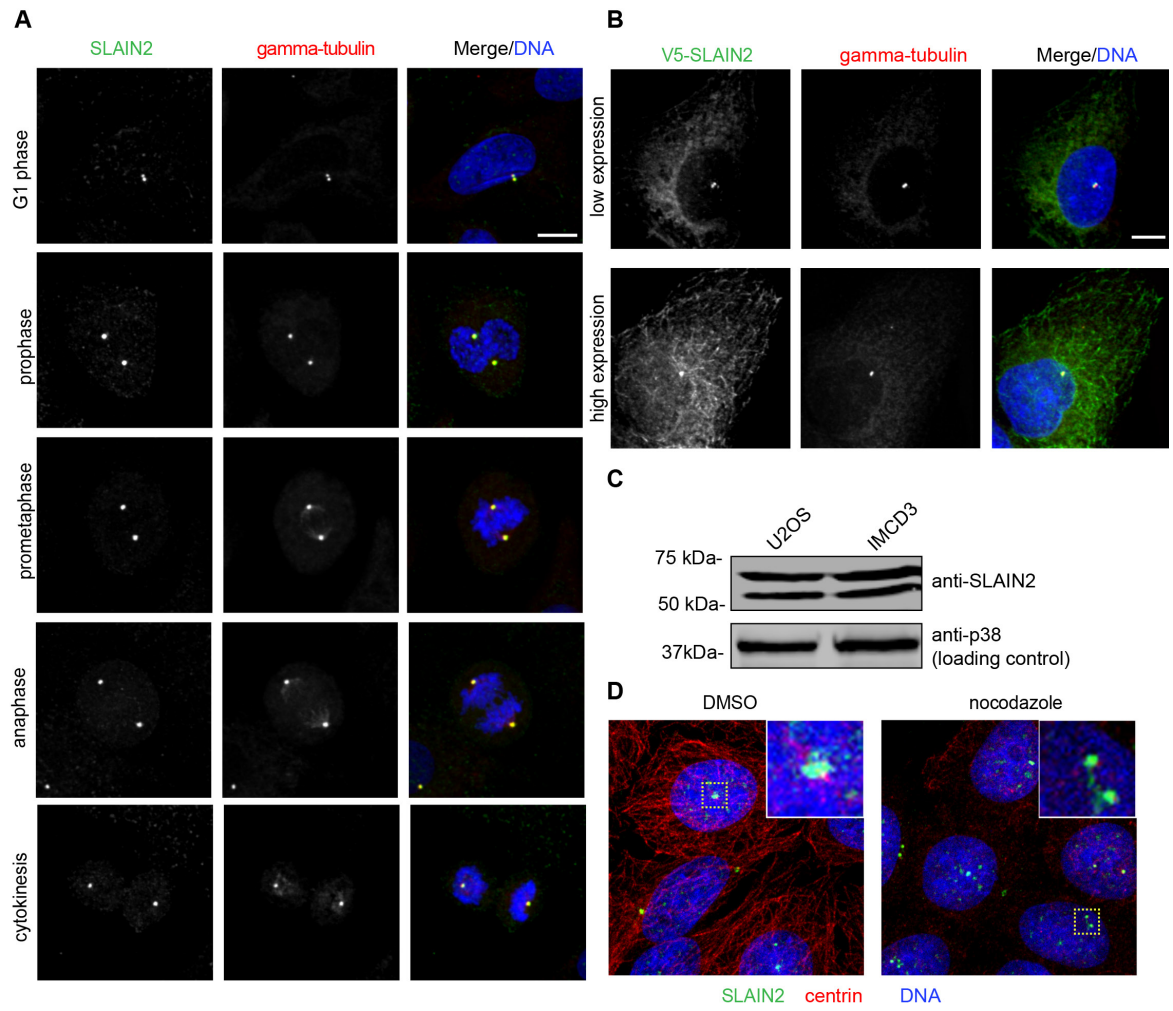
SLAIN2 localizes to the centrosome. (A) Asynchronous U2OS cells were immunostained with antibodies against SLAIN2 (green) and gamma-tubulin (red), which marks the pericentriolar material. DNA was stained with DAPI. Representative images of SLAIN2 localization at different stages of the cell cycle are shown. (B) V5-SLAIN2 fusion protein localizes to the centrosome in lowexpressing cells and to the centrosome and microtubules in high-expressing cells. U2OS heterogeneous pools stably expressing V5-SLAIN2 were immunostained with antibodies against V5 and gamma-tubulin. (C) Immunoblots of human U2OS and mouse IMCD3 cell extracts with anti-SLAIN2 antibody and anti-p38 antibody, which was used a loading control. The expected size for full length SLAIN2 is 62 kDa, which corresponds to the upper band. The lower band likely represents an isoform of SLAIN2. (D) SLAIN2 localizes to the centrosome upon microtubule depolymerization. U2OS cells were treated with DMSO vehicle control or nocodazole for 1 h and immunostained with antibodies against SLAIN2 and alpha-tubulin, which marks the centrosome. The yellow dashed boxes represent the centrosomes used for zoomed insets. Scale bars represent 10 μm.

To investigate the cellular functions and mechanisms of SLAIN2 at the centrosome, we examined the localization of endogenous SLAIN2 and a V5-SLAIN2 fusion protein in human osteosarcoma U2OS cells, which are well-characterized for their centrosome biogenesis and ideal for visualizing the microtubule network due to their flat morphology. We determined endogenous SLAIN2 localization by staining cells with a rabbit polyclonal antibody raised against the C-terminal 231-581 amino acid fragment of SLAIN2 relative to staining for the centrosome marker gamma-tubulin. The different cell cycle phases were distinguished by the distinct staining of the nucleus. SLAIN2 colocalized with gamma-tubulin at all cell cycle stages, apparent as centrosome staining as one or two proximate puncta next to the nucleus in interphase cells and spindle pole staining from early prophase to telophase in mitotic cells (Figure 1A). Analogous to endogenous SLAIN2, V5-SLAIN2 colocalized with gamma-tubulin in U2OS cells stably expressing the fusion protein (Figure 1B). Notably, V5-SLAIN2 also localized to the microtubule ends high expressing cells (Figure 1B), which is consistent with its reported characterization as microtubule plus-end protein (van der Vaart et al., 2011). Of note, microtubule tip localization was not apparent in U2OS cells immunostained with anti-SLAIN2 antibody. Supporting its specificity, anti-SLAIN2 antibody recognized a specific band at the expected size of 581 amino acid isoform 1 (62.5 kDa) in U2OS and mouse inner medullary collecting duct IMCD3 cell extracts by Western blotting (Figure 1C). There was also an additional smaller band, which likely represents a different SLAIN2 isoform. 

Given the affinity of SLAIN2 to microtubules, we next tested whether SLAIN2 localization to the centrosome depends on microtubules or not. To this end, U2OS cells were treated with nocodazole to depolymerize microtubules, which was confirmed by immunostaining for alpha-tubulin. SLAIN2 localized to the centrosomes in both cells treated with DMSO vehicle control and nocodazole, suggesting that centrosomal localization of a fraction of SLAIN2 at the centrosome is independent of microtubules (Figure 1D). Collectively, these results identify SLAIN2 as a bona fide centrosome protein and suggest functions for SLAIN2 at the centrosome in addition to its reported functions at the microtubule plus ends. 

### 3.2. SLAIN2 localizes to the proximal end of the centrioles

**Figure 2:**
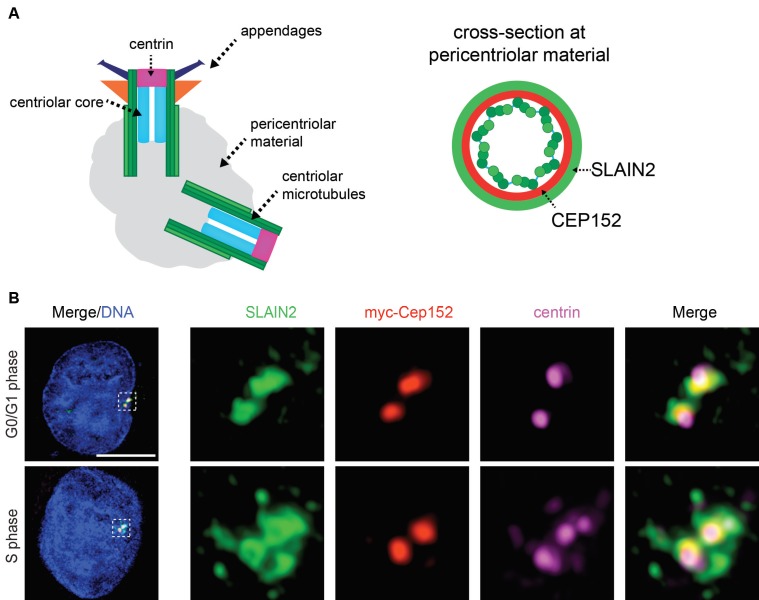
SLAIN2 localizes to the proximal end of the centrioles at the pericentriolar material. (A) Schematic representation of different subcentrosomal domains with their associated markers. Cross-section view of the proximal end of centrioles represents the localization of SLAIN2 at the pericentriolar material relative to Cep152. (B) U2OS cells were transfected with myc-Cep152 expression construct, fixed and stained for SLAIN2 (green), myc-Cep152 (pericentriolar material protein) (red) and centrin 3 (distal end of centrioles) (magenta). DNA was stained with DAPI (blue). For high-resolution imaging, fluorescence signals were detected with HyD detectors and processed with Hyvolution software. Representative images for SLAIN2 localization in G0/G1 phase and S phase cells (duplicated centrioles) are shown. Scale bars represent 5 μm. Insets show 6X enlarged centrosomes.

Centrosomes are composed of two centrioles and associated pericentriolar material. Application of superresolution microscopy approaches to centrosome proteins over the years have revealed a highly regulated architecture for the centrosome and distinct subcentrosomal domains were associated with specific functions (Figure 2A) (Luders 2012). To gain insight into the centrosomal functions of SLAIN2, we used high resolution imaging and defined the spatial localization of SLAIN2 at the centrosome. To this end, SLAIN2 was immunostained with the centriole distal end marker Centrin 3 and the pericentriolar material component Cep152 (Sonnen et al., 2012). SLAIN2 localized to a resolvable ring at the proximal end of the centrioles in both G0/G1 and S phase cells, which encompassed Cep152 staining at the pericentriolar material (Figures 2B and S1). Confirming its proximal localization, SLAIN2 and centrin signals did not overlap (Figure S1). These results identify SLAIN2 as a component of the pericentriolar material at the proximal end of centrioles and suggest functions associated with this subdomain such as microtubule dynamics, pericentriolar material organization, and/or centriole duplication. 

### 3.3. Gene ontology and STRING analysis of the SLAIN2 proximity interactome 

**Figure 3:**
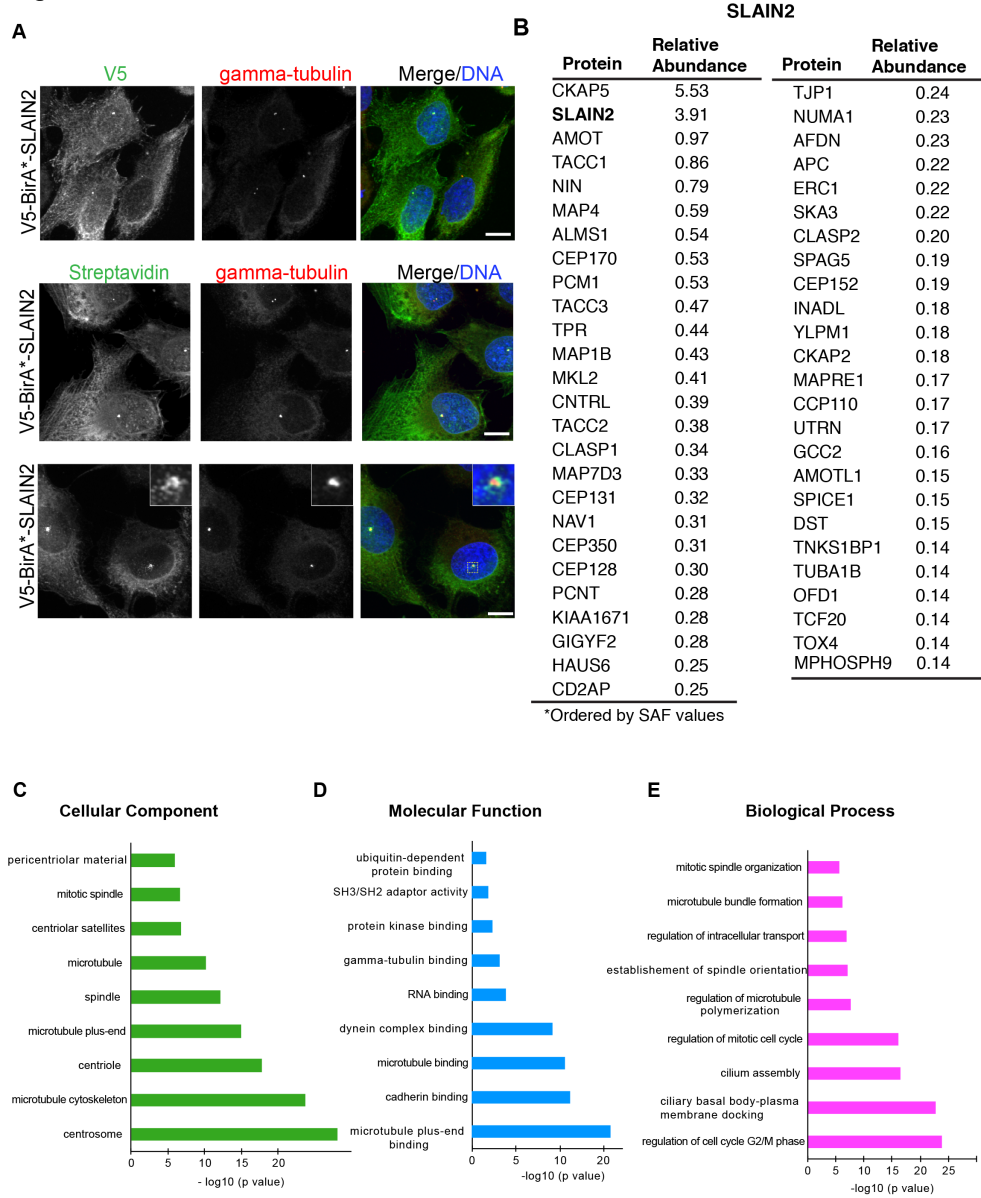
Localization, activity and proximity interactors of BirA*-SLAIN2. (A) HEK293T cells stably expressing BirA-SLAIN2 were incubated with 50 μM biotin for 18 h, fixed and stained for V5-BirA*-SLAIN2 with V5 antibody, biotinylated proteins with fluorescent streptavidin and centrosomes with anti-gamma-tubulin antibody. Scale bars represent 10 μm. Insets show 4× enlarged centrosomes. (B) Mass spectrometry analysis of proximity interactors of BirA*-SLAIN2. The top 50 proximity interactors are ranked in the order of their spectral counts normalized to the total spectral count of each mass spectrometry dataset generated from three experimental replicates. Bolded proteins were previously shown to interact physically with SLAIN2. (C, D, E) Gene ontology analysis of the top 100 proximity interactors of SLAIN2 based on (C) cellular compartment, (D) molecular function, and (E) biological process. GO analysis was performed using EnrichR. The bar graphs indicate the –log 10 of P-values for the indicated GO terms.

BioID-based proximity labeling has proven to be a powerful tool in defining interactions at the centrosome, in particular the transient highly dynamic or insoluble ones (Firat-Karalar and Stearns, 2015). To gain insight into the cellular functions of SLAIN2 in an unbiased way, we identified proximity interactors of SLAIN2 using the BioID approach. Myc-BirA (R118G) (hereafter BirA*) was fused to the N-terminus of SLAIN2 in a lentiviral expression vector, which was used to generate HEK293T cells stably expressing BirA*-SLAIN2. After 18 h of incubation of the cells with 50 µM biotin, we found that BirA*-SLAIN2 localized to the centrosome and induced biotinylation at the centrosome, as assessed by streptavidin staining to detect biotinylated proteins and the centrosome marker anti-gamma-tubulin (Figure 3A). In addition to its localization at the centrosome, BirA*-SLAIN2 also localized to and induced biotinylation at the microtubules (Figure 3A). Of note, we note that V5 signal of the ectopically expressed fusion protein was specific to the microtubule plus ends, the biotinylation signal was detected along microtubule filaments. This difference is likely due to covalent biotinylation of the spatial proximity of BirA* at the microtubules during 18 h of biotinylation. Finally, in a small subset of cells, BirA*-SLAIN2 induced biotinylation of granules around the centrosome, which likely are the centriolar satellites (Figure 3A). These results confirm correct localization of the V5-BirA*-SLAIN2 fusion protein at the centrosome and microtubules. 

To identify proximity interactors of SLAIN2, we performed large-scale streptavidin pulldowns from HEK293T::BirA*-SLAIN2 and HEK293T::BirA* cells. To this end, the cells were incubated with 50 µM biotin for 18 h and lysed by denaturing lysis buffer to solubilize the centrosome. Biotinylated proteins were pulled down by streptavidin chromatography and analyzed by mass spectrometry. HEK293T cells stably expressing BirA* itself was processed in parallel as a control. We analyzed the mass spectrometry data from three experimental replicates for BirA*-SLAIN2 and seven experimental replicates for BirA* by using a label-free quantitative proteomics approach termed normalized spectral abundance factor (NSAF) (Zybailov et al., 2006). For NSAF analysis, we accounted for run-to-run variation by comparing spectral counts of each identified protein with the total spectral count of the run. Of note, we did not normalize the data with the length of identified proteins. After filtering out nonspecific proteins based on comparative analysis of SLAIN2 datasets with control datasets (>2.5 fold enrichment) and common mass spectrometry contaminants, we identified 167 high-confidence proximity interactions for BirA*-SLAIN2. 

The top 50 proximity interactors for SLAIN2 are listed in Figure 3B. The SLAIN2 proximity interactome includes the components of the SLAIN2 complex at the microtubule plus-ends including CLASP1/2, EB1/2, and CKAP5 (van der Vaart et al., 2011). Additionally, we compared the SLAIN2 proximity interactome with the list of putative SLAIN2 interactors that we derived from BioGRID, a curated repository for protein interaction datasets that includes mass spectrometry data generated by affinity purification, yeast two hybrid screen, and proximity labeling approaches (Stark et al., 2006). This comparison identified CKAP5, SH3KBP1, CEP170, CEP128, CNTRL, PCM1, TACC3, TACC2, CEP131, MAPRE1, and MAPRE2 as the shared interactors, which identifies them as high confidence SLAIN2 interactors. Importantly, 156 interactions were specific to SLAIN2 proximity interactome. 

For a more global and quantitative analysis of the SLAIN2 proximity interactome, we performed gene ontology (GO) analysis for the top 100 proximity interactors of SLAIN2 based on cellular compartment, molecular function, and biological process. GO analysis for cellular compartment identified “centrosome”, “microtubule cytoskeleton”, and “spindle” as highly enriched compartments, which is in agreement with localization of SLAIN2 to the centrosome and microtubules (Figure 3C). GO analysis showed that in molecular function terms, the upregulated functions were highly enriched in “microtubule plus-end binding”, “cadherin binding”, and “dynein complex binding” (Figure 3D). While the association with microtubules and dynein is in agreement with its reported microtubule-associated functions, its proximity to cadherin-binding proteins suggests possible functions for SLAIN2 in cell adhesion. Interestingly, “RNA binding”, “gamma-tubulin binding”, “protein kinase binding”, “SH3/SH2 adaptor activity”, and “ubiquiting-dependent protein binding” were also enriched in the interactome. Finally, in biological terms, “regulation of cell cycle”, “cilium assembly” and “regulation of intracellular transport” were highly enriched (Figure 3E). In order to reveal the interactions between the proximity interactors of SLAIN2, we organized the top 100 proximity interactors into a protein interaction network using the STRING database (Figure S2). This interaction network revealed “centrosomes” and “microtubules” as the main hubs. 

### 3.4. SLAIN2 has extensive interactions with centrosome and microtubule-associated proteins

**Figure 4:**
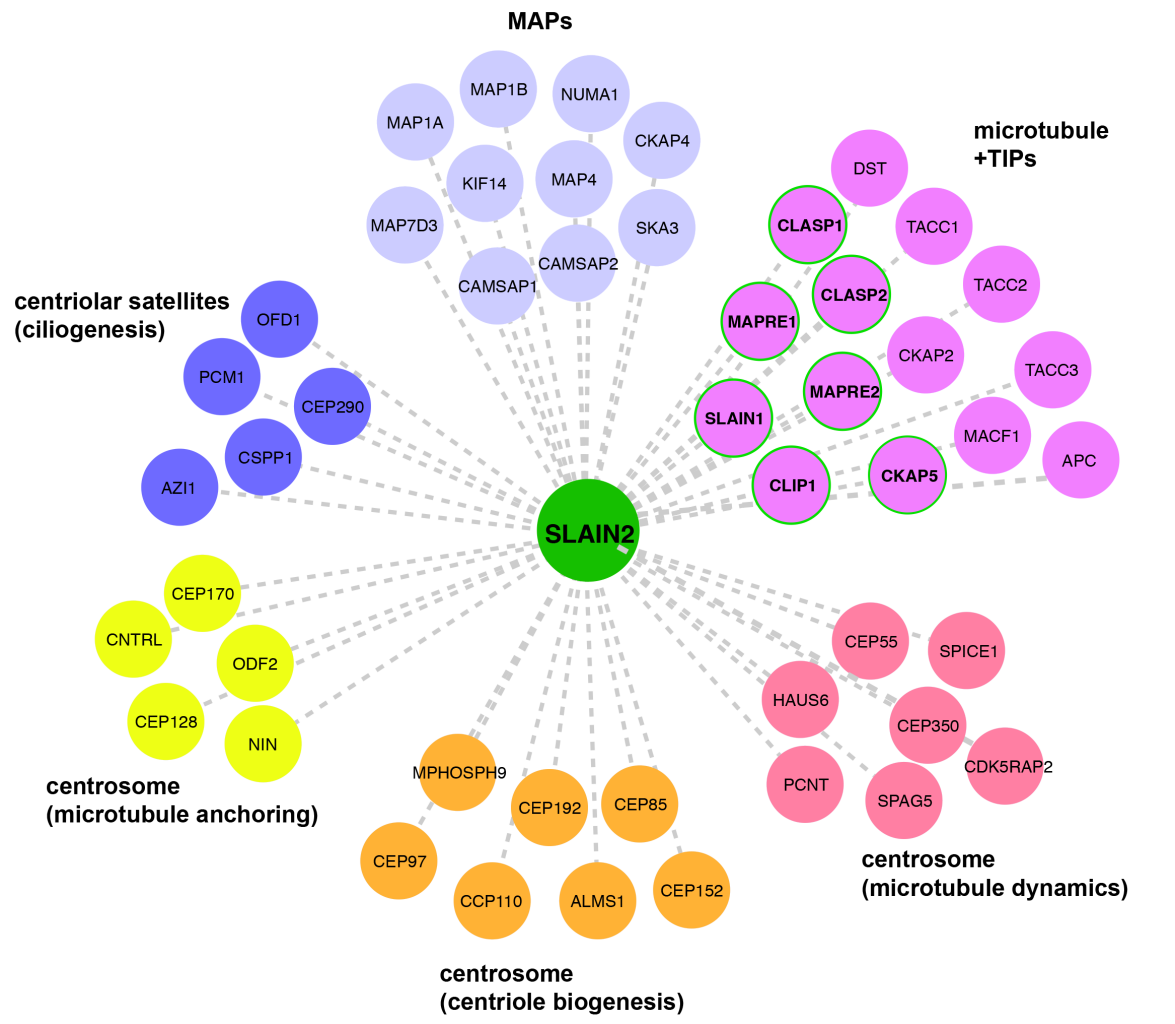
SLAIN2 proximity interaction map at the centrosome and microtubules. The proximity interaction map of SLAIN2 was constructed with selected proteins from mass spectrometry analysis using the Cytoscape software. The edges are color-coded according to their function and localization. The magenta edges with green periphery represent the proximity interactors of SLAIN2 that were also identified as physical interactors by previous studies. MAP: microtubule-associated protein, +TIP: microtubule plus-end binding protein.

To gain further insight into the functions of SLAIN2 at the centrosome, we mined the group of novel SLAIN2 interactors and manually created a list of highly abundant SLAIN2 interactors with well-defined functions in previous studies. Among the new SLAIN2 interactors, multiple clusters stand out by their functions in key cellular processes associated with centrosomes, cilia, and microtubules. We manually organized these proteins according to their previously described functions and generated a network map of SLAIN2 using Cytoscape (Figure 4). Moreover, we generated a protein interaction network among these proteins using the STRING database (Figure S3). Proteins that were previously defined by their localization and functions at the microtubule plus ends were highly enriched in the SLAIN2 interactome, which is in agreement with the well-characterized +TIP localization of SLAIN2 (Akhmanova and Steinmetz, 2008; Akhmanova and Steinmetz, 2010). Strikingly, CKAP5 was the most abundant +TIP protein in the proximity map, even more abundant than SLAIN2 itself. Given that BioID-mediated biotinylation is proximity-dependent, the identification of CKAP5 as the top prey suggests a stable and/or long-lived interaction between CKAP5 and SLAIN2. In addition to +TIPs, SLAIN2 have highly abundant proximity interactions to other microtubule-associated proteins (MAPs), including microtubule minus-end-binding proteins CAMSAPs (Jiang et al., 2014). 

Another cluster that stood out in SLAIN2 proximity maps is the group of centrosome and centriolar satellite proteins. Consistent with our characterization of SLAIN2 as a component of the pericentriolar material, SLAIN2 interacts with a wide range of pericentriolar material proteins that were previously identified as regulators of centriole duplication and mitosis. Moreover, SLAIN2 has proximity interactions with a group of centriolar satellite proteins including PCM1, AZI1/CEP131, and OFD1, which is consistent with biotinylation of satellites in a subset of BirA*-SLAIN2-expressing cells and suggests functions for SLAIN2 during cilium assembly (Odabasi et al., 2019; Prosser and Pelletier, 2020). Finally, a subset of subdistal appendage proteins such as NIN and CEP128 were identified in SLAIN2 proximity maps (Uzbekov and Alieva, 2018). Notably, NIN was among the first five proximity interactors of SLAIN2. Given the function of subdistal appendages in anchoring microtubules at the centrosome, this relationship suggests centrosomal functions for SLAIN2 in organization of microtubules. 

## 4. Discussion

SLAIN2 is a microtubule plus-end tracking protein implicated in cancer. Here we used the BioID approach to generate the first in vivo proximity map of SLAIN2. Our results provide new insight into the functions and mechanisms of SLAIN2 through identification of its proximity to previously characterized proteins and complexes at the centrosomes and microtubules. Of note, our results identify SLAIN2 as a dual regulator of centrosome- and microtubule associated functions and show that SLAIN2 mediates its functions in a more complex mechanism than the one proposed by previous studies. 

The proximity interactome of SLAIN2 included most known physical interactions at the microtubule tips reported by previous studies (Akhmanova and Steinmetz, 2010). Interestingly, 50 of the putative SLAIN2 interactors curated from the BioGRID database was not identified in the SLAIN2 interactome. This could be in part due to the differences in the nature of interactions revealed by proximity-mapping, affinity purifications and yeast two hybrid experiments or due to the interference of the N-terminal BirA*-tag between SLAIN2 and a subset of its interactors such as ch-TOG. In fact, a direct comparison of affinity purifications and proximity mapping showed that these two approaches identify both overlapping and distinct interactions and thus should be used as complementary approaches to define the complete set of interactors for proteins of interest (Lambert et al., 2014). 

 A striking feature of the SLAIN2 proximity map is that it is highly enriched for well-characterized centrosome proteins. This is in agreement with our identification of SLAIN2 as a bona fide centrosome protein that localizes to the proximal end of centrioles. We note that despite our identification of SLAIN2 at the centrosomes of mitotic cells, a previous study showed that SLAIN2 loses its association with microtubules upon phosphorylation in mitosis (van der Vaart et al., 2011). Among the centrosome proteins proximate to SLAIN2, several functional modules stand out by their link to cellular processes that go awry in cancer. Centriole duplication is a highly regulated process that ensures duplication of centrioles once during cell cycle. CEP152, CEP192, and CEP85 function in initiation of centriole duplication by regulating centriolar recruitment of the kinase PLK4 (Nigg and Holland, 2018). CEP97 and CCP110 cap the distal ends of centrioles and regulate centriole length (Spektor et al., 2007; Schmidt et al., 2009). Deregulation of centriole duplication and length results in supernumerary centrioles, which is a hallmark for cancer and thus this mechanism might explain the link between SLAIN2 and cancer (Nigg and Raff, 2009; Godinho et al., 2014; Marteil et al., 2018;). In addition to centriole duplication, centriolar satellites proteins are also abundant in the SLAIN2 proximity interactome (Prosser and Pelletier 2020). Among these proteins are PCM1, CEP290, CSPP1, AZI1/CEP131, and OFD1, which are well-characterized as key regulators of primary cilium assembly and function (Firat-Karalar, 2018; Prosser and Pelletier, 2020). Finally, consistent with the identification of SLAIN2 as a component of pericentriolar material by high-resolution imaging, SLAIN2 has proximity interactions with pericentriolar material proteins such as CEP55 and SPAG5 that function in cell division, and PCNT, CDK5RAP2, and CEP250 that regulate pericentriolar material organization. Future studies are required to define the specific centrosomal functions of SLAIN2. 

The detection of an interactor by BioID-mediated biotinylation correlates with its proximity to the BirA*-fusion protein, the time spent in the vicinity of the BirA*-fusion protein and its cellular abundance. Given that we performed BioID pulldowns in cells stably expressing BirA*-SLAIN2 fusions, the resulting proximity interactome is a good resource for predicting the potential direct interactors of SLAIN2 as well as the transient ones. We predict that the top proximity interactors identified in the SLAIN2 dataset are likely its direct interactors, which is supported by the identification of +TIPs including CKAP5, TACC1/2/3, and CLASP1 among the top 10 interactors. In addition to +TIPs, centriolar satellite (PCM1, CEP131), subdistal appendage (NIN, CEP170), and pericentriolar material proteins (ALMS1) are also among this highly abundant group, suggesting functions for SLAIN2 at these subcentrosomal domains. Although the relative abundance of preys is a good starting point for predicting the functionally relevant SLAIN2 interactors, we note that they should not be used alone since a subset of critical interactions might be missed due to their transient and low abundance nature. 

The in vivo SLAIN2 proximity interactome we generated provides a powerful resource for future studies aimed at dissecting SLAIN2 functions and mechanisms at the centrosome. An important first step to investigate these functions is the confirmation of the putative SLAIN2 interactions using a set of different approaches. To assay which proximity interactions are reflected at the physical level, coimmunoprecipitation experiments can be used. However, given the nature of the proximity labeling approach, the set of insoluble and/or transient interactions will not be confirmed by this conventional method. A good alternative to overcome the limitations of conventional methods is the proximity ligation assay, which allows in situ visualization of proteins within 30 nm of each other (Bagchi et al., 2015). Once the SLAIN2 interactions are validated, testable hypothesis about its functions could be generated and tested in subsequent loss-of-function and gain-of-function experiments. 

## Acknowledgments

I acknowledge Şevket Onur Taflan, Melis Dilara Arslanhan, and Ebru Topcu for insightful discussions regarding this work. I also acknowledge use of the services and facilities of the Koç University Proteomics Facility. This work was supported by EMBO Installation Grant, TÜBİTAK Grant 115Z521, FABED Eser Tümen Research Award, and Turkish Academy of Sciences Distinguished Young Scientist Award to ENF.

## Supplementary Material

**Figure S1**Subcentrosomal localization of SLAIN2 relative to CEP152 and centrin. U2OS cells were fixed and stained with the indicated antibodies and images were acquired with Leica SP8 Hyvolution confocal. Intensity plots of SLAIN2, myc-CEP152, and centrin for the top-view are generated by the “Plot Profile” tool in ImageJ. Scale bar 0.5 μm.
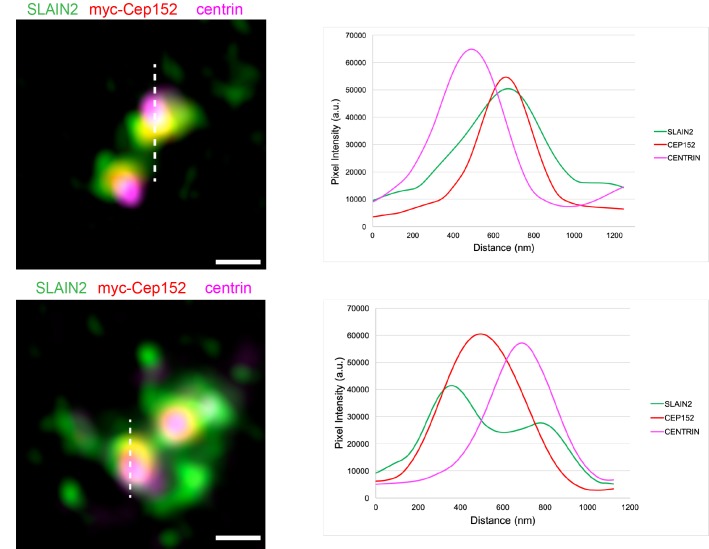

**Figure S2**STRING interaction network for the top 100 proximity interactors of SLAIN2. Known and predicted interactions between the top 100 proximity interactors of SLAIN2 according to the STRING database.
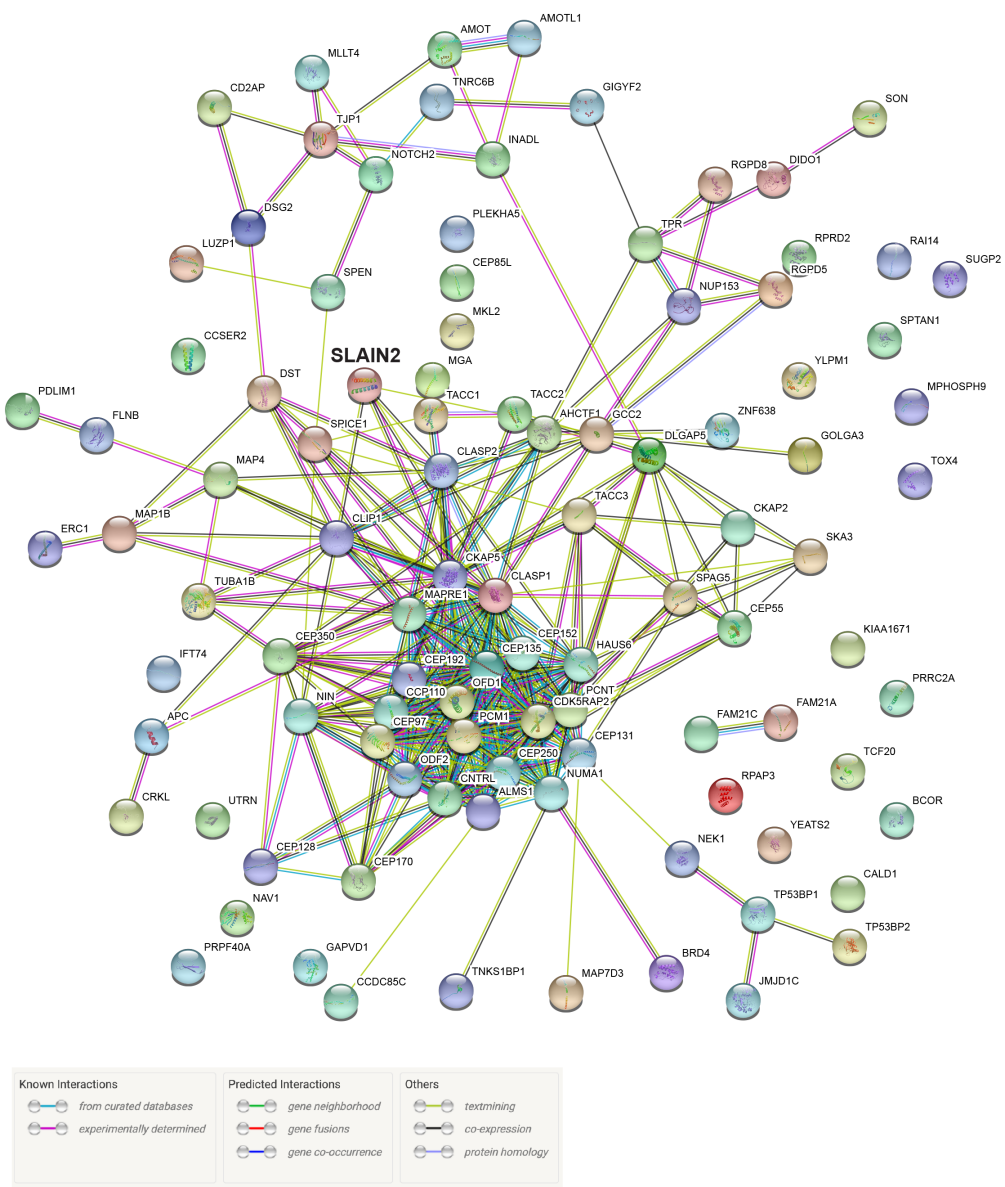

**Figure S3**STRING interaction network for the proximity interactors of SLAIN2 implicated in centrosome- and microtubuleassociated functions. Known and predicted interactions between the selected proximity interactors of SLAIN2 according to the STRING database.